# Unsupervised phenotyping of suspect keratoconus based on posterior tomography and epithelial remodeling

**DOI:** 10.1186/s12886-026-05084-1

**Published:** 2026-06-27

**Authors:** Abdelrahman Salman

**Affiliations:** Al-Mashrek Laser Vision Center, Tartous, Syria

**Keywords:** Suspect keratoconus, Keratoconus, Epithelial remodeling, Scheimpflug imaging, Sirius tomography, AS-OCT

## Abstract

**Purpose:**

To characterize structural heterogeneity within suspect keratoconus (SKC) using an unsupervised phenotyping strategy integrating posterior tomographic and epithelial remodeling parameters.

**Methods:**

In this cross-sectional study, 55 eyes with clinically defined SKC underwent Scheimpflug–Placido tomography and anterior segment optical coherence tomography. Posterior asymmetry indices (Symmetry Index Back [SIb] and Keratoconus Vertex Back [KVb]) and epithelial redistribution metrics (Minimum–Maximum epithelial thickness difference [Min–Max ET] and Superonasal–Inferotemporal epithelial thickness difference [SN–IT ET], 2–5 mm zone) were entered into k-means clustering without predefined diagnostic thresholds. Demographic and refractive variables were compared between derived phenotypes. Independent anterior surface elevation and keratometric parameters were analyzed for structural validation. Cluster robustness and multidimensional separation within the SKC cohort were assessed using silhouette analysis, principal component analysis (PCA), posterior-only sensitivity analysis, and hierarchical clustering sensitivity analysis.

**Results:**

Two distinct structural phenotypes emerged. A posterior-asymmetric phenotype (26 eyes) demonstrated significantly higher SIb and KVb (both *p* < 0.001) together with greater epithelial redistribution (*p* ≤ 0.002). A second phenotype (29 eyes) exhibited comparatively milder structural alterations. No significant differences were observed in age, sex distribution, visual acuity, refractive error, or minimum corneal thickness (all *p* > 0.05). Simulated keratometric indices (K1, K2, Kavg) did not distinguish between phenotypes, whereas anterior elevation parameters differed significantly. PCA loading analysis and posterior-only sensitivity analysis supported posterior tomographic abnormalities as the principal axis of phenotype separation. The two-cluster solution showed a silhouette coefficient of 0.29, and hierarchical clustering sensitivity analysis supported its robustness (agreement = 87.5%, Cohen’s κ = 0.729).

**Conclusions:**

Suspect keratoconus is not a structurally uniform entity but comprises distinct structural phenotypes driven principally by posterior tomographic asymmetry and accompanied by epithelial remodeling. Epithelial remodeling provided complementary structural information rather than serving as the dominant driver of cluster separation. Because longitudinal and biomechanical data were unavailable, the future clinical significance and progression risk of these phenotypes remain unknown and require further investigation.

**Supplementary Information:**

The online version contains supplementary material available at 10.1186/s12886-026-05084-1.

## Introduction

Keratoconus (KC) is a progressive ectatic corneal disorder characterized by stromal thinning, protrusion, and progressive visual deterioration [1]. Early detection remains critical, particularly in refractive surgery candidates, in whom undiagnosed subclinical disease may predispose to postoperative ectasia [2, 3]. While the diagnosis of clinically manifest KC is generally straightforward, identifying suspect keratoconus (SKC) represents a persistent diagnostic challenge [4].

Over the past decade, advances in Scheimpflug-based tomography and anterior segment optical coherence tomography (AS-OCT) have substantially improved early ectasia detection. Posterior corneal surface indices and pachymetric progression metrics demonstrated high diagnostic accuracy in differentiating SKC from normal corneas [5, 6]. Similarly, epithelial thickness mapping has emerged as a complementary modality capable of revealing compensatory epithelial remodeling patterns that may mask underlying stromal irregularity [7–9].

Despite these advances, most studies including our previous work, have focused primarily on diagnostic discrimination, aiming to distinguish SKC from normal eyes using threshold-based approaches or multivariate models [4, 8, 9, 10]. This paradigm implicitly assumes that SKC represents a relatively homogeneous preclinical stage along a linear continuum toward KC. However, increasing evidence suggests that early ectatic disease may not follow a uniform structural trajectory. Variability in posterior deformation, epithelial compensation patterns, and tomographic asymmetry may indicate the presence of distinct structural phenotypes within what is currently labeled as “suspect keratoconus” [11, 12]. Whether SKC represents a single entity or a spectrum of structurally heterogeneous subtypes remains unclear.

Understanding such heterogeneity is scientifically and clinically relevant. If structurally distinct SKC phenotypes exist, they may represent different structural states within the early ectatic spectrum. However, whether these phenotypes differ in biomechanical behavior, progression risk, surgical suitability, or clinical outcomes remains unknown and requires longitudinal investigation. Moreover, reliance on conventional keratometric parameters alone may obscure meaningful structural divergence occurring at the posterior corneal surface or within epithelial redistribution patterns.

To address this gap, the present study adopts an unsupervised phenotyping approach to investigate structural heterogeneity within a cohort of SKC eyes. By integrating posterior tomographic indices and epithelial thickness remodeling parameters without predefined diagnostic thresholds, we aim to determine whether distinct structural subtypes can be identified within SKC and to characterize the relative contribution of posterior asymmetry and epithelial compensation to this heterogeneity.

We hypothesize that SKC is not a structurally uniform condition but rather comprises distinct phenotypic subsets driven primarily by posterior tomographic alterations, with epithelial remodeling reflecting complementary compensatory responses.

## Materials and methods

### Study design and ethical approval

This retrospective observational study was conducted at Al-Mashrek Laser Vision Center and adhered to the tenets of the Declaration of Helsinki. The study protocol was approved by the Institutional Review Board of Al-Mashrek Laser Vision Center. Previously collected clinical data were analyzed in anonymized form, and the requirement for written informed consent was waived.

### Participants

Eyes with a prior clinical diagnosis of SKC were eligible for inclusion. Eyes were classified as SKC when they demonstrated no slit-lamp biomicroscopic or retinoscopic signs of KC, maintained corrected distance visual acuity (CDVA) ≥ 1.0 (20/20), and were categorized as SKC by the integrated Sirius Scheimpflug–Placido topographic/tomographic classifier [10]. All SKC classifications were established before the present clustering analysis was performed.

Inclusion was based on this composite clinical–tomographic diagnosis rather than on any individual clustering variable. Although posterior tomographic indices may contribute to the integrated Sirius classification framework, epithelial thickness mapping was not part of the diagnostic classification process [13]. Therefore, study inclusion was not determined by the clustering variables themselves or by predefined thresholds of individual study parameters.

Exclusion criteria included clinical KC, prior ocular surgery (including refractive surgery or corneal cross-linking), corneal scarring or other corneal pathology, active ocular disease, contact lens wear within the required discontinuation period, incomplete records, poor-quality scans, pregnancy, and systemic connective tissue disorders.

**Fig. 1:**
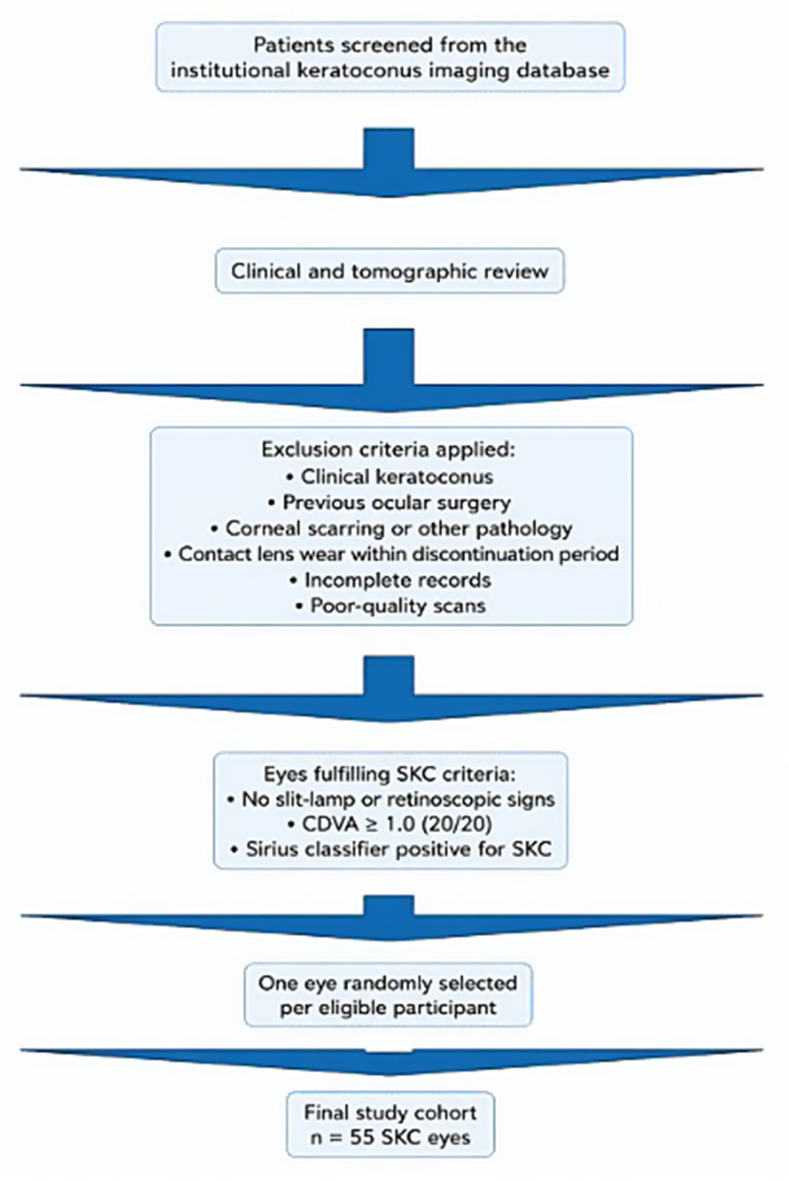
Study flowchart and SKC eligibility criteria. Patient flowchart showing database screening, eligibility assessment, exclusion criteria, SKC diagnostic criteria, random eye selection, and final study cohort. SKC criteria required absence of slit-lamp or retinoscopic signs of keratoconus, CDVA ≥ 1.0 (20/20), and positive Sirius classifier categorization for SKC

In addition to the primary SKC cohort, a separate set of normal corneas and eyes with established KC was used exclusively for comparative visualization and structural contextualization. These eyes were not included in the clustering procedure used to derive SKC phenotypes. Figure 1 summarizes the patient screening process, exclusion criteria, SKC eligibility criteria, random eye selection, and final study cohort.

### Imaging protocol

All included eyes underwent standardized corneal imaging using: Scheimpflug–Placido corneal tomography (Sirius, CSO, Florence, Italy), and AS-OCT epithelial thickness mapping (Cirrus 5000, Carl Zeiss Meditec, Germany). Only scans meeting manufacturer-recommended quality criteria were included in the analysis. No missing data were present for the variables included in the clustering and sensitivity analyses; eyes with incomplete datasets or poor-quality tomographic or epithelial scans were excluded before analysis.

### Tomographic and epithelial parameters

The following tomographic indices were extracted: Symmetry Index Back (SIb), reflecting inferior–superior asymmetry of the posterior corneal surface; Keratoconus Vertex Back (KVb), representing maximal posterior elevation relative to the reference surface.

Minimum corneal thickness (ThkMin), Symmetry Index Front (SIf), and Simulated keratometry values; flat keratometry (K1), steep keratometry (K2), and average keratometry (Kavg).

Epithelial remodeling was assessed using: Minimum–Maximum epithelial thickness difference (Min–Max ET) within the 2–5 mm paracentral zone, Superonasal–Inferotemporal epithelial thickness difference (SN–IT ET) within the 2–5 mm zone.

Min–Max ET was calculated as the arithmetic difference between the minimum and maximum epithelial thickness values within the defined zone.

### Unsupervised structural phenotyping

To investigate structural heterogeneity within SKC, an unsupervised clustering approach was implemented. Four structural variables were selected a priori for clustering based on their established diagnostic relevance in early ectatic disease detection and their ability to capture posterior and epithelial structural characteristics [8, 10]: posterior corneal asymmetry was quantified using SIb, posterior elevation abnormality using KVb, magnitude of epithelial redistribution using Min–Max ET within the 2–5 mm paracentral zone, and directional epithelial asymmetry using SN–IT ET within the same zone.

Anterior surface symmetry (SIf) and keratometric parameters (K1, K2, Kavg) were intentionally excluded from clustering to avoid curvature-driven grouping and to permit independent evaluation of anterior surface characteristics across derived phenotypes.

Prior to clustering, variables were standardized using z-score normalization to account for differences in measurement scales. K-means clustering was selected because the study objective was exploratory phenotyping using continuous standardized structural variables and because centroid-based partitioning provides a parsimonious and interpretable method for identifying structural subgroups. The number of clusters was evaluated using silhouette analysis in conjunction with clinical interpretability. Although the silhouette coefficient was marginally higher for k = 3 than k = 2 (0.31 vs. 0.29), k = 2 was selected as the final solution because it provided greater parsimony, avoided over-partitioning of the modest sample into a small potentially unstable subgroup, and yielded clinically interpretable phenotypes. The robustness of the two-phenotype solution was further evaluated using posterior-only and hierarchical clustering sensitivity analyses.

For the posterior-only sensitivity analysis, k-means clustering was repeated using only SIb and KVb, and the resulting assignments were compared with the original four-variable model. For the hierarchical clustering sensitivity analysis, Ward’s agglomerative clustering with Euclidean distance was performed using the same four standardized clustering variables as the primary model. Agreement, Cohen’s kappa coefficient, and adjusted Rand index were calculated where applicable after aligning arbitrary cluster labels.

### Principal component analysis

Principal component analysis (PCA) was performed to explore the multidimensional structural relationships within SKC and to visualize the spatial distribution of derived phenotypes. PCA was performed using the four clustering variables (SIb, KVb, Min–Max ET [2–5 mm], and SN–IT ET [2–5 mm]) together with minimum corneal thickness (ThkMin).

ThkMin was intentionally excluded from the clustering procedure to avoid potential bias toward global pachymetric effects and to ensure that phenotype derivation was driven primarily by posterior asymmetry and epithelial redistribution patterns. However, it was included in the PCA to evaluate its contribution to overall structural variance and to assess its relationship with posterior and epithelial parameters within the multidimensional space.

All variables were standardized using z-score normalization prior to analysis. PCA was constructed using only the SKC cohort to characterize internal structural variance. Normal corneas and clinically manifest KC eyes were subsequently projected onto the PCA space for contextual visualization and were not used in component derivation.

The first two principal components were retained for graphical representation, explaining 53.8% and 16.8% of the total variance, respectively. PCA was used exclusively as a dimensionality-reduction and visualization technique; it did not influence cluster formation and was not interpreted as proof of discrete biological classes.

### Statistical analysis

All statistical analyses were performed using R version 4.5.2 (R Foundation for Statistical Computing, Vienna, Austria). Continuous variables were assessed for normality using the Shapiro–Wilk test. As the majority of variables were not normally distributed, data are presented as median (interquartile range). Between-group comparisons were performed using the Mann–Whitney U test for continuous variables. Categorical variables were compared using the chi-square test or Fisher’s exact test, as appropriate. Spearman correlation analysis was performed to evaluate associations between posterior tomographic abnormalities and epithelial remodeling parameters. Standardized Euclidean distances were calculated from standardized median structural profiles to contextualize SKC phenotypes relative to normal and KC eyes. All statistical tests were two-tailed, and a p-value < 0.05 was considered statistically significant.

## Results

### Cohort characteristics and phenotype identification

A total of 55 eyes diagnosed with SKC were included in the phenotyping analysis. Unsupervised k-means clustering identified two structural phenotypes: Phenotype 0 (*n* = 26) and Phenotype 1 (*n* = 29).

The silhouette coefficient was 0.29 for k = 2 and 0.31 for k = 3. Although k = 3 showed a marginally higher silhouette value, the two-phenotype solution was retained based on parsimony, clinical interpretability, and additional sensitivity analyses.

As shown in Table 1, no statistically significant differences were observed between phenotypes in age, sex distribution, uncorrected distance visual acuity (UDVA), CDVA, sphere, cylinder, or spherical equivalent (all *p* > 0.05). These findings indicate that phenotype separation was not driven by demographic or refractive characteristics.

**Table 1:** Baseline demographic and refractive characteristics of SKC phenotypes

Variable	Phenotype 0 (*n* = 26)	Phenotype 1 (*n* = 29)	*p*-value	Cliff’s δ
Sex (F/M), n	10/16	14/15	0.588	
Age (years)	23.50 [18.00, 30.25]	25.00 [20.00, 32.00]	0.374	-0.14
UDVA (decimal)	0.50 [0.40, 0.70]	0.60 [0.50, 0.80]	0.329	-0.19
CDVA (decimal)	1.00 [1.00, 1.00]	1.00 [1.00, 1.00]	0.805	-0.03
Sphere (D)	0.00 [-0.69, 0.00]	0.00 [-0.38, 0.25]	0.874	-0.03
Cylinder (D)	-1.75 [-2.69, -1.00]	-0.75 [-2.63, -0.25]	0.098	-0.27
Spherical equivalent (D)	-1.00 [-1.97, -0.41]	-0.75 [-1.44, -0.11]	0.274	-0.18

UDVA, uncorrected distance visual acuity; CDVA, corrected distance visual acuity; D, diopters. Continuous variables are presented as median [interquartile range]. Between-group comparisons were performed using the Mann–Whitney U test for continuous variables and Fisher’s exact test for categorical variables. Effect size is reported as Cliff’s delta (δ)

### Core structural parameters used for clustering

The four structural variables used for clustering are summarized in Table 2. Phenotype 0 demonstrated significantly greater posterior corneal asymmetry and elevation than Phenotype 1. SIb was significantly higher in Phenotype 0 than in Phenotype 1 (0.78 [0.61–0.89] vs. 0.29 [0.20–0.41], *p* < 0.001, Cliff’s delta = 0.77), and KVb was also substantially higher (31.00 [23.25–33.75] µm vs. 14.77 [11.00–19.00] µm, *p* < 0.001, Cliff’s delta = 0.91).

**Table 2:** Posterior tomographic and epithelial remodeling parameters used for unsupervised SKC phenotyping

Variable	Phenotype 0 (*n* = 26)	Phenotype 1 (*n* = 29)	*p*-value	Cliff’s δ
SIb (D)	0.78 [0.61, 0.89]	0.29 [0.20, 0.41]	< 0.001	0.77
KVb (µm)	31.00 [23.25, 33.75]	14.77 [11.00, 19.00]	< 0.001	0.91
ThkMin (µm)	475.50 [445.75, 508.75]	486.00 [471.00, 512.00]	0.191	-0.21
Min–Max ET (2–5 mm) (µm)	-12.00 [-13.75, -10.00]	-9.00 [-11.00, -7.00]	0.002	-0.48
SN–IT ET (2–5 mm) (µm)	4.00 [3.00, 6.00]	0.00 [-1.00, 1.00]	< 0.001	0.81

SIb, Symmetry Index Back; D, diopters; KVb, Keratoconus Vertex Back; µm, micrometers; ThkMin, minimum corneal thickness; Min–Max ET (2–5 mm), minimum minus maximum epithelial thickness within the 2–5 mm paracentral zone; SN–IT ET (2–5 mm), superonasal minus inferotemporal epithelial thickness within the 2–5 mm zone

Variables were standardized using z-score normalization prior to k-means clustering. Continuous variables are presented as median [interquartile range]. Mann–Whitney U test was used for between-group comparisons. Effect size is reported as Cliff’s delta (δ)

Minimum corneal thickness did not differ significantly between phenotypes (475.50 [445.75–508.75] µm vs. 486.00 [471.00–512.00] µm, *p* = 0.191), indicating that global pachymetric thinning was not the primary feature separating the clusters.

Epithelial remodeling parameters also differed significantly between groups. Min–Max ET (2–5 mm) was more negative in Phenotype 0 than in Phenotype 1 (− 12.00 [− 13.75 to − 10.00] µm vs. − 9.00 [− 11.00 to − 7.00] µm, *p* = 0.002, Cliff’s delta = − 0.48), indicating greater epithelial redistribution. Directional epithelial asymmetry, represented by SN–IT ET (2–5 mm), was also significantly greater in Phenotype 0 (4.00 [3.00–6.00] µm vs. 0.00 [− 1.00 to 1.00] µm, *p* < 0.001, Cliff’s delta = 0.81).

### Independent anterior surface and keratometric validation

Independent anterior-surface and keratometric parameters not included in the clustering model are presented in Table 3. SIf and KVf were significantly higher in Phenotype 0 than in Phenotype 1 (SIf: 2.02 [1.35–2.52] vs. 0.81 [0.49–1.02], *p* < 0.001, Cliff’s delta = 0.65; KVf: 12.00 [10.00–14.75] µm vs. 6.00 [4.00–8.39] µm, *p* < 0.001, Cliff’s delta = 0.74).

**Table 3:** Anterior surface and keratometric parameters for independent validation of derived SKC phenotypes

Variable	Phenotype 0 (*n* = 26)	Phenotype 1 (*n* = 29)	*p*-value	Cliff’s δ
K1 (D)	42.24 [41.61, 43.80]	42.67 [41.89, 43.78]	0.639	-0.08
K2 (D)	45.28 [43.89, 45.97]	44.37 [43.49, 45.40]	0.163	0.22
Kavg (D)	43.67 [42.68, 44.68]	43.87 [42.68, 44.34]	0.683	0.07
SIf (D)	2.02 [1.35, 2.52]	0.81 [0.49, 1.02]	< 0.001	0.65
KVf (µm)	12.00 [10.00, 14.75]	6.00 [4.00, 8.39]	< 0.001	0.74

K1, flat keratometry; D, diopters; K2, steep keratometry; Kavg, average keratometry; SIf, Symmetry Index Front; KVf, Keratoconus Vertex Front; µm, micrometers. Variables were not included in the clustering model and are presented for independent validation. Continuous variables are presented as median [interquartile range]. Mann–Whitney U test was used for comparisons. Effect size is reported as Cliff’s delta (δ)

In contrast, simulated keratometric curvature parameters did not differ significantly between phenotypes. K1, K2, and Kavg were comparable between groups (all *p* > 0.05). These findings indicate that anterior elevation and asymmetry reflected structural divergence, whereas conventional curvature-based keratometric measures remained comparable.

### Posterior–epithelial correlation analysis

Correlations between posterior tomographic abnormalities and epithelial remodeling parameters are shown in Table 4. A strong positive correlation was observed between SIb and KVb (*r* = 0.812, *p* < 0.001), confirming substantial co-variation between posterior asymmetry and posterior elevation.

**Table 4:** Correlations between posterior tomographic abnormalities and epithelial remodeling parameters within the SKC cohort

Variable Pair	Spearman’s *r*	*p*-value
SIb vs. KVb	0.812	< 0.001
SIb vs. Min–Max ET (2–5 mm)	-0.295	0.027
SIb vs. SN–IT ET (2–5 mm)	0.345	0.009
KVb vs. Min–Max ET (2–5 mm)	-0.358	0.007
KVb vs. SN–IT ET (2–5 mm)	0.504	< 0.001
Min–Max ET (2–5 mm) vs. SN–IT ET (2–5 mm)	-0.189	0.162

SKC, suspect keratoconus; SIb, Symmetry Index Back; KVb, Keratoconus Vertex Back; Min–Max ET (2–5 mm), minimum minus maximum epithelial thickness difference within the 2–5 mm paracentral zone; SN–IT ET (2–5 mm), superonasal minus inferotemporal epithelial thickness difference within the 2–5 mm paracentral zone. Spearman correlation coefficients were calculated to evaluate the relationship between posterior tomographic abnormalities and epithelial remodeling parameters within the SKC

Correlations between posterior tomographic indices and epithelial remodeling parameters were weak to moderate. SIb correlated with Min–Max ET (*r* = − 0.295, *p* = 0.027) and SN–IT ET (*r* = 0.345, *p* = 0.009). KVb correlated with Min–Max ET (*r* = − 0.358, *p* = 0.007) and SN–IT ET (*r* = 0.504, *p* < 0.001). Min–Max ET and SN–IT ET were not significantly correlated with each other (*r* = − 0.189, *p* = 0.162).

These findings indicate that epithelial remodeling was associated with posterior structural abnormality but did not simply duplicate posterior tomographic information, supporting its role as complementary rather than redundant structural information.

### Principal component analysis and PCA loadings

**Fig. 2:**
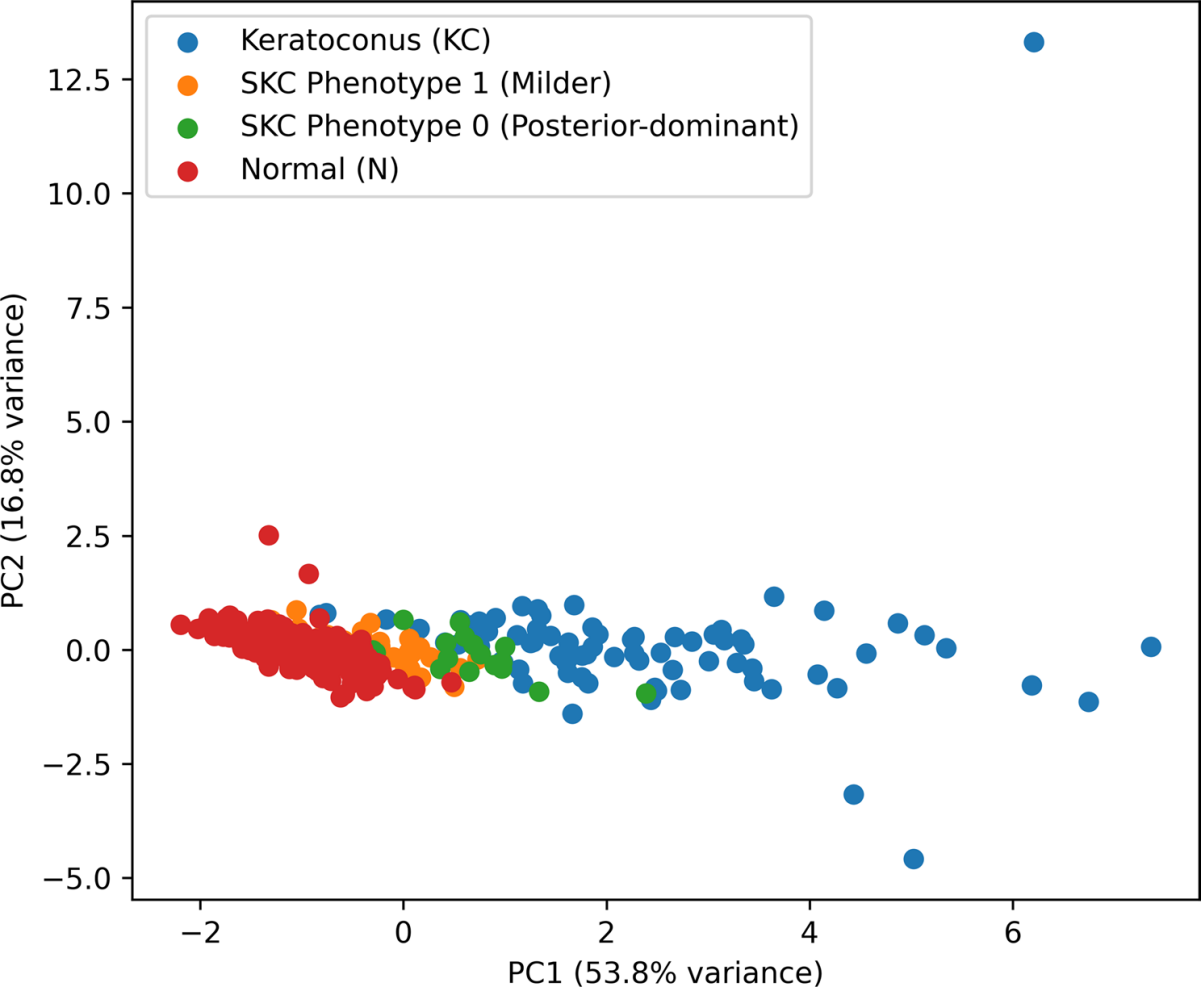
Principal component analysis (PCA) positioning plot for Normal (N), Suspect Keratoconus (SKC) phenotypes, and Keratoconus (KC) eyes based on the five structural variables (SIb, KVb, ThkMin, Min–Max ET [2–5 mm], SN–IT ET [2–5 mm]). PC1 explains 53.8% of the total variance and PC2 explains 16.8%. PCA was used solely as a dimensionality-reduction and visualization technique and did not contribute to cluster generation or establish discrete biological classes

Principal component analysis (PCA) was performed using the four clustering variables together with ThkMin to visualize multidimensional structural separation. As shown in Fig. 2, the first two principal components explained 53.8% and 16.8% of the total variance, respectively, accounting collectively for 70.6% of overall variability. Partial but consistent spatial separation was observed between Phenotype 0 and Phenotype 1. PCA was used only for visualization and did not influence cluster assignment.

PCA loading analysis is presented in Table 5. Posterior tomographic variables contributed most strongly to PC1, with KVb and SIb showing the highest loadings (0.625 and 0.585, respectively). Epithelial remodeling variables showed intermediate contributions, whereas ThkMin contributed predominantly to PC2. These findings support posterior tomographic asymmetry as the principal structural axis underlying phenotype separation.

**Table 5:** Principal component loadings of structural variables included in the PCA model

Variable	PC1 Loading	PC2 Loading
SIb (D)	0.585	0.179
KVb (µm)	0.625	0.132
Min–Max ET (2–5 mm) (µm)	-0.221	-0.481
SN–IT ET (2–5 mm) (µm)	0.426	-0.342
ThkMin (µm)	-0.191	0.776

PCA, principal component analysis; SIb, Symmetry Index Back; D, diopter, KVb, Keratoconus Vertex Back; Min–Max ET (2–5 mm), µm minimum minus maximum epithelial thickness difference within the 2–5 mm paracentral zone; SN–IT ET (2–5 mm), superonasal minus inferotemporal epithelial thickness difference within the 2–5 mm paracentral zone; ThkMin, minimum corneal thickness. Higher absolute loading values indicate greater contribution to each component

### Structural contextualization relative to normal and keratoconus eyes

To contextualize the two SKC phenotypes relative to normal and keratoconic eyes, standardized Euclidean distances were calculated using median structural profiles across SIb, KVb, ThkMin, Min–Max ET (2–5 mm), and SN–IT ET (2–5 mm), as shown in Table 6.

**Table 6:** Standardized structural distances between normal corneas, SKC phenotypes, and KC

Comparison	Standardized Euclidean Distance
Normal vs. Phenotype 1	0.858
Normal vs. Phenotype 0	1.600
Phenotype 0 vs. Phenotype 1	1.056
Phenotype 0 vs. KC	1.619
Phenotype 1 vs. KC	2.630
Normal vs. KC	3.174

SKC, suspect keratoconus; KC, keratoconus. Standardized Euclidean distances were calculated from standardized median structural profiles. Lower values indicate greater structural proximity between groups

Phenotype 1 was closer to normal corneas than Phenotype 0 (distance: 0.858 vs. 1.600). Conversely, Phenotype 0 was closer to KC than Phenotype 1 (distance: 1.619 vs. 2.630). These findings suggest that the two SKC phenotypes occupy different structural positions along the continuum between normal corneas and clinically manifest KC.

### Cluster profile and sensitivity analyses

**Fig. 3:**
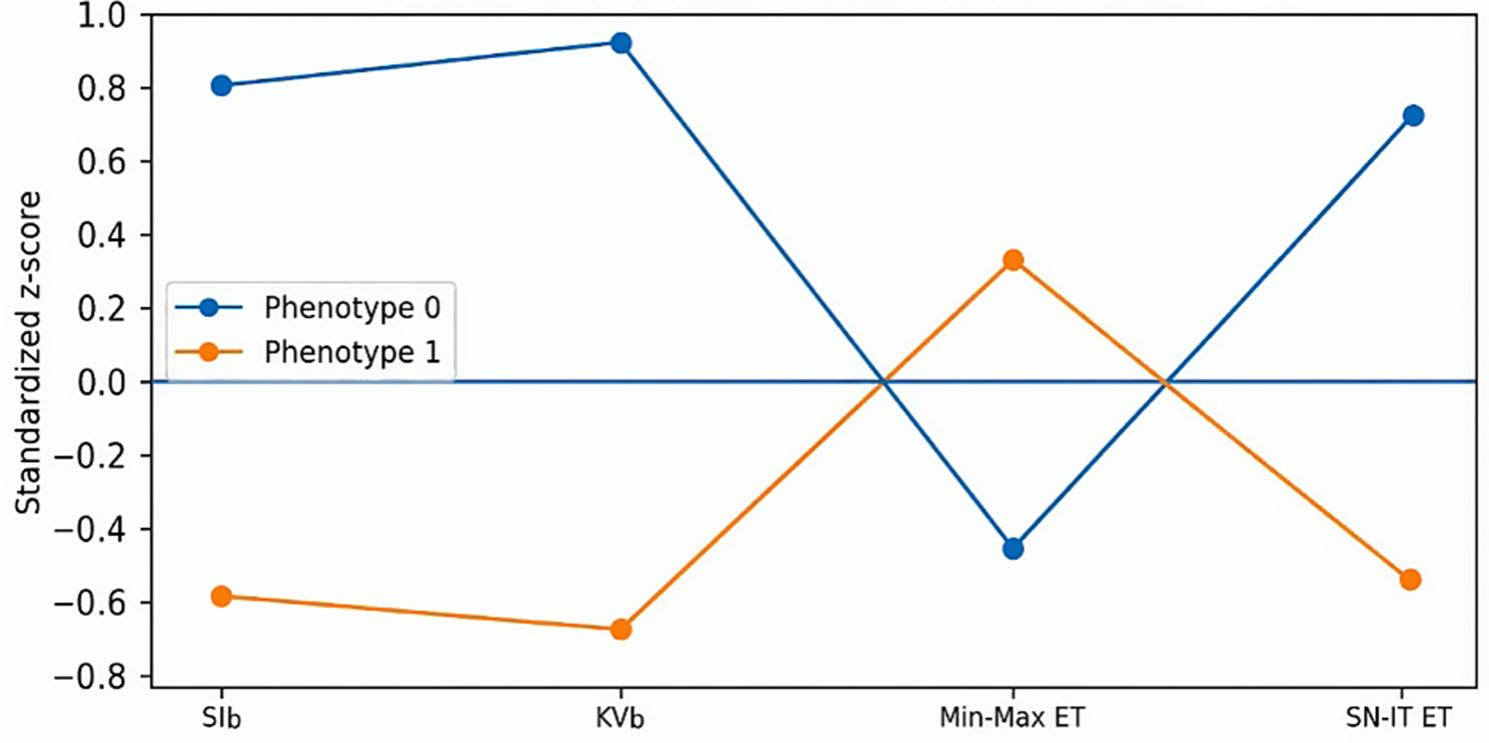
Standardized structural profiles of SKC phenotypes across the four clustering variables. Phenotype 0 (*n* = 26) demonstrates higher posterior asymmetry/elevation (SIb and KVb) and greater directional epithelial asymmetry (SN–IT ET). Because Min–Max ET is defined as minimum minus maximum epithelial thickness, more negative values indicate greater epithelial redistribution; therefore, the lower standardized score for this variable in Phenotype 0 reflects more pronounced epithelial remodeling

The standardized cluster profile plot is shown in Fig. 3. Phenotype 0 demonstrated higher standardized scores for SIb, KVb, and SN–IT ET, together with a lower standardized score for Min–Max ET. Because Min–Max ET is defined as minimum minus maximum epithelial thickness, more negative values indicate greater epithelial redistribution. Therefore, the lower standardized value for Min–Max ET in Phenotype 0 reflects more pronounced epithelial remodeling rather than less structural abnormality.

Posterior-only clustering sensitivity analysis using SIb and KVb alone demonstrated 85.5% agreement with the original four-variable clustering solution (Cohen’s kappa = 0.690; Supplementary Table S1). This finding indicates that posterior tomographic abnormalities constituted the principal driver of phenotype separation, whereas epithelial variables provided complementary discriminatory information.

As an additional sensitivity analysis, Ward’s hierarchical clustering was performed using the same four standardized clustering variables. When partitioned into two clusters, the hierarchical solution demonstrated high concordance with the primary k-means model (agreement = 87.5%, Cohen’s kappa = 0.729, adjusted Rand index = 0.553; Supplementary Table S2 and Supplementary Figure S1). The hierarchical k = 2 solution yielded a silhouette coefficient of 0.378.

Together, these sensitivity analyses support the robustness and reproducibility of the two-phenotype structure and further justify retention of k = 2 as the primary clustering solution despite the marginally higher silhouette coefficient observed for k = 3.

## Discussion

The present study demonstrates that SKC does not represent a structurally homogeneous preclinical stage, but rather comprises distinct structural phenotypes primarily defined by posterior corneal asymmetry and associated epithelial remodeling. By applying an unsupervised clustering framework independent of predefined diagnostic thresholds, we identified two structural subgroups within clinically defined SKC eyes. These findings support a reinterpretation of SKC not as a uniform preclinical entity, but as a continuum of structural states within the early ectatic spectrum.

Cluster separation was predominantly driven by posterior asymmetry and elevation indices (SIb and KVb), whereas anterior curvature parameters (K1, K2, and Kavg) did not significantly distinguish between phenotypes. This pattern reinforces accumulating evidence that early ectatic alterations preferentially involve the posterior corneal surface before manifest anterior curvature changes become detectable [10, 14, 15]. The present sensitivity analyses further support posterior tomography as the principal axis of phenotype separation, while epithelial remodeling provides complementary structural information. From a biomechanical perspective, posterior surface irregularity may reflect subtle stromal weakening that precedes clinically apparent anterior steepening, partially masked by epithelial compensation [16].

Our previous work demonstrated that SIb was the most sensitive Sirius-derived parameter for differentiating SKC from normal corneas [10]. The present findings extend this observation by showing that SIb not only discriminates SKC from normal eyes but also stratifies structural variability within SKC itself. This supports posterior asymmetry as a core structural axis of early ectatic heterogeneity rather than merely a diagnostic discriminator [17]. These results are consistent with prior studies emphasizing posterior elevation and asymmetry in subclinical KC detection [13, 18, 19]. However, unlike supervised classifiers designed to maximize diagnostic separation, our unsupervised framework interrogates intrinsic structural architecture, thereby revealing latent heterogeneity within a clinically uniform SKC cohort.

Posterior-only clustering based exclusively on SIb and KVb reproduced most of the original four-variable classification, with 85.5% agreement and a Cohen’s κ of 0.690. The additional hierarchical clustering sensitivity analysis also supported the robustness of the two-phenotype structure. Although the silhouette coefficient was modest and k = 3 showed a marginally higher silhouette value than k = 2, Ward’s hierarchical clustering using the same four standardized variables showed high concordance with the primary k-means solution (agreement = 87.5%, Cohen’s κ = 0.729, adjusted Rand index = 0.553). This supports the stability of the two-phenotype structure and justifies retaining k = 2 as a parsimonious and clinically interpretable solution in this modest-sized cohort.

Although epithelial redistribution parameters contributed to cluster formation, the present analyses consistently indicated that posterior tomographic abnormalities represented the dominant structural axis underlying phenotype separation. Posterior-only sensitivity analysis reproduced most of the original clustering solution (agreement = 85.5%, Cohen’s κ = 0.690), and PCA loading analysis demonstrated stronger contributions from posterior variables (KVb and SIb) than from epithelial remodeling metrics. These findings suggest that epithelial remodeling reflects secondary structural adaptation accompanying posterior abnormalities rather than serving as the principal discriminator of SKC phenotypes. This interpretation is consistent with the epithelial masking hypothesis proposed by Reinstein and colleagues [20–22].

In the present cohort, epithelial redistribution differed significantly between phenotypes and remained associated with posterior asymmetry patterns. However, posterior–epithelial correlations were only weak to moderate, indicating that epithelial changes did not fully account for phenotype separation. These findings align with our previous work demonstrating superior diagnostic performance of posterior tomographic indices compared with epithelial thickness–based parameters for detecting suspect keratoconus [8, 9]. Therefore, epithelial mapping appears to provide additional structural context but lower discriminatory contribution than posterior tomographic assessment in early ectatic characterization.

Correlation analysis demonstrated a strong association between SIb and KVb, confirming that posterior asymmetry and posterior elevation co-varied within the SKC cohort. In contrast, correlations between posterior tomographic indices and epithelial redistribution metrics were only weak-to-moderate. Although significant correlations were observed between several posterior and epithelial parameters, none of the posterior–epithelial correlations were sufficiently strong to suggest collinearity or justify exclusion of epithelial metrics from the structural phenotyping model. Accordingly, epithelial mapping should be interpreted as complementary structural information rather than as an inferior or redundant modality.

The absence of significant differences in simulated keratometric parameters (K1, K2, and Kavg) between phenotypes underscores an important limitation of curvature-based assessment in early ectatic disease. Despite clear structural divergence at the posterior surface and epithelial level, anterior curvature metrics remained comparable across subgroups. However, this should not be interpreted as evidence that all anterior-derived indices are uninformative. SIf and KVf, which reflect anterior asymmetry and elevation/irregularity, differed significantly between phenotypes. In our previous Sirius-based diagnostic study, SIb demonstrated the highest ability to detect SKC (AUC = 0.863), exceeding SIf (AUC = 0.791), KVf (AUC = 0.704), average keratometry (AUC = 0.542), and Curve-Apex (AUC = 0.568) [10]. Thus, the present findings should be interpreted as showing relative insensitivity of conventional curvature-based keratometry, rather than lack of value of anterior assessment in general. Whether structurally distinct SKC phenotypes differ in future clinical behavior remains unknown and requires longitudinal investigation.

Minimum corneal thickness did not differ significantly between phenotypes, indicating that global thinning was not the principal axis of structural divergence within this SKC cohort. Although pachymetric reduction is a recognized hallmark of established KC [23], our findings suggest that in the subclinical stage, asymmetric posterior elevation and surface irregularity may precede measurable differences in minimum thickness. This observation reinforces the concept that early ectatic remodeling may be characterized more by spatial redistribution and asymmetry than by absolute thinning [24, 25]. Accordingly, reliance on minimum pachymetry alone may overlook meaningful structural variability within SKC.

The identification of structurally distinct phenotypes within clinically defined SKC has important implications for structural characterization of early ectatic disease. If SKC represents a spectrum of structural states rather than a uniform preclinical entity, multidimensional imaging may provide a more informative description of early corneal abnormalities than conventional keratometry alone. However, the present findings should be interpreted as structural phenotyping rather than clinical risk stratification. No longitudinal follow-up or biomechanical data were available, and the future clinical behavior of either phenotype remains unknown. Although it is plausible that posterior-asymmetric phenotypes may differ in biomechanical properties or progression trajectories, this remains a hypothesis requiring prospective longitudinal and biomechanical validation.

From a research perspective, our results emphasize the value of unsupervised analytical approaches in uncovering latent structural heterogeneity within clinically defined categories. Traditional supervised models optimize discrimination between predefined groups; however, they may obscure internal variability within diagnostic labels. By interrogating intrinsic structural architecture, unsupervised clustering provides complementary insight into disease heterogeneity and may help generate hypotheses for future longitudinal studies. Integrating corneal biomechanics, longitudinal tomographic changes, and genetic markers may further clarify whether posterior-dominant phenotypes represent distinct structural states within the ectatic continuum.

Several limitations should be acknowledged. First, the cross-sectional design precludes causal inference regarding the temporal relationship between posterior asymmetry and epithelial remodeling and does not allow assessment of progression risk. Second, longitudinal follow-up and biomechanical measurements were not available; therefore, whether either phenotype is earlier, faster, biomechanically weaker, or clinically higher risk remains unknown. Third, the sample size was modest and derived from a single center, which limits the ability to explore more granular subgroup structures and requires external validation. Fourth, clustering stability was moderate by silhouette analysis, reflecting partial overlap between phenotypes and supporting interpretation of SKC as a structural spectrum rather than two completely discrete biological classes. Fifth, PCA was used only as a visualization and dimensionality-reduction tool and should not be interpreted as proof of discrete biological entities. Sixth, all measurements were device-specific and derived from Sirius tomography and Cirrus epithelial mapping; replication across other platforms is necessary to confirm generalizability. Finally, although poor-quality scans and incomplete records were excluded, retrospective datasets may still be subject to selection bias.

In conclusion, suspect keratoconus does not constitute a structurally homogeneous entity. Within clinically defined SKC, we identified two structural phenotypes primarily differentiated by posterior tomographic asymmetry and associated epithelial remodeling, while conventional anterior curvature metrics and minimum pachymetry remained comparable. Posterior tomographic abnormalities, particularly SIb and KVb, constituted the principal axis of phenotype separation, whereas epithelial remodeling provided complementary structural information. Because longitudinal and biomechanical data were unavailable, the future clinical significance and progression risk of these phenotypes remain unknown and warrant prospective validation.

## Supplementary Information

Below is the link to the electronic supplementary material.

Supplementary Material 1

## Data Availability

The datasets generated and/or analyzed during the current study are available from the corresponding author on reasonable request.
