# Supplementary material for: Unsupervised phenotyping of suspect keratoconus based on posterior tomography and epithelial remodeling
Source: BMC Ophthalmol. 2026 Jun 27;26:357. doi: 10.1186/s12886-026-05084-1 (PMC13312653; doi:10.1186/s12886-026-05084-1)
Supplement: Supplementary file 1 — Supplementary Material 1 [file 12886_2026_5084_MOESM1_ESM.docx]

**Supplementary Material for: Unsupervised Phenotyping of Suspect Keratoconus Based on Posterior Tomography and Epithelial Remodeling**

**Table S1.** Posterior-only sensitivity analysis using SIb and KVb.

| **Metric** | **Value** |
| --- | --- |
| Agreement with original four-variable clustering (%) | 85.5 |
| Cohen’s κ coefficient | 0.690 |

SIb, Symmetry Index Back; KVb, Keratoconus Vertex Back; κ, Cohen’s kappa coefficient. Posterior-only clustering was performed using SIb and KVb. Agreement with the original four-variable clustering solution was calculated after alignment of cluster labels. The high concordance suggests that posterior tomographic variation contributed substantially to phenotype separation.

**Table S2.** Hierarchical clustering sensitivity analysis using the same four standardized variables employed in the primary k-means model.

| **Metric** | **Value** |
| --- | --- |
| Clustering variables | SIb, KVb, Min–Max ET (2–5 mm), SN–IT ET (2–5 mm) |
| Standardization | Z-score normalization |
| Hierarchical method | Ward’s agglomerative clustering using Euclidean distance |
| Number of clusters evaluated | k = 2 |
| Agreement with original four-variable k-means clustering after label alignment (%) | 87.5 |
| Cohen’s κ coefficient | 0.729 |
| Adjusted Rand Index | 0.553 |
| Silhouette coefficient of hierarchical k = 2 solution | 0.378 |

SIb, Symmetry Index Back; KVb, Keratoconus Vertex Back; Min–Max ET, minimum minus maximum epithelial thickness difference; SN–IT ET, superonasal minus inferotemporal epithelial thickness difference; κ, Cohen’s kappa coefficient. High concordance with the original clustering solution supports the robustness of the two-phenotype structure.


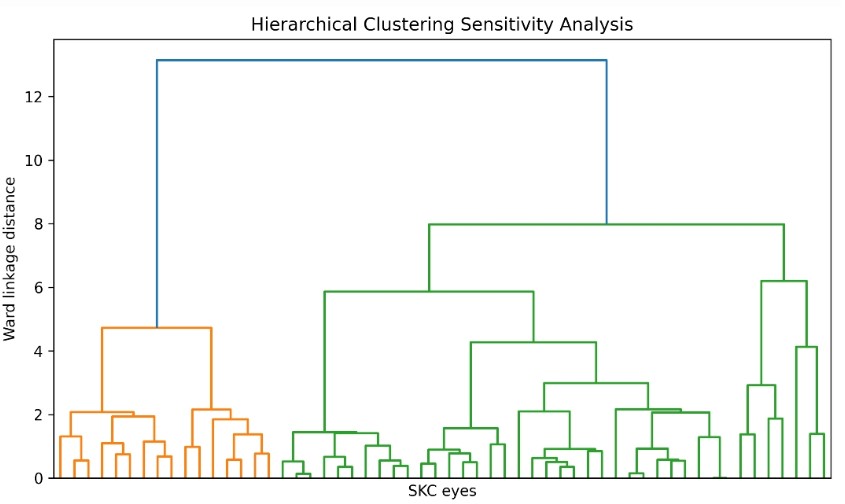


**Figure S1.** Hierarchical clustering sensitivity analysis performed using the same four standardized structural variables employed in the primary k-means model: Symmetry Index Back (SIb), Keratoconus Vertex Back (KVb), Min–Max epithelial thickness difference (2–5 mm), and superonasal–inferotemporal epithelial thickness asymmetry (SN–IT ET, 2–5 mm).

Ward’s agglomerative clustering with Euclidean distance was applied. The dendrogram demonstrates two major structural groupings. When partitioned into two clusters, the hierarchical solution showed high concordance with original k-means clustering, supporting the robustness of the two-phenotype solution.
